# Naphthenic Acid-Induced ROS Emissions by Rainbow Trout Mitochondria

**DOI:** 10.3390/toxics13121015

**Published:** 2025-11-24

**Authors:** Zahra Kalvani, Pius Tetteh, Collins Kamunde, Don Stevens, Michael R. van den Heuvel

**Affiliations:** 1 Department of Biomedical Sciences, Atlantic Veterinary College, University of Prince Edward Island, Charlottetown, PE C1A 4P3, Canada; patetteh@upei.ca (P.T.); ckamunde@upei.ca (C.K.); dstevens@upei.ca (D.S.); 2 Department of Biology, Canadian Rivers Institute, University of Prince Edward Island, Charlottetown, PE C1A 4P3, Canada; mheuvel@upei.ca

**Keywords:** naphthenic acid fraction compounds, oil sands process-affected water, oil sands, reactive oxygen species, hydrogen peroxide, mitochondria, rainbow trout, *Oncorhynchus mykiss*, Oroboros Oxygraph-2k high-resolution respirometer

## Abstract

Naphthenic acid fraction compounds (NAFC) are prevalent in petrochemical wastewater, including from oil sands processing, and induce reactive oxygen species (ROS) emissions in isolated mitochondria. The purpose of this study was to verify if a primary carboxylic acid, the moderately hydrophobic NAFC 3,5-dimethyladamantane-1-acetic acid, would cause the mitochondrial ROS (hydrogen peroxide; H_2_O_2_) production and affect its consumption by mitochondria in multiple bioenergetic states. Intact mitochondria isolated from rainbow trout (*Oncorhynchus mykiss*) liver were exposed to commercially available 3,5-dimethyladamantane-1-acetic acid. The emission of ROS during States 3 and 4 respiration was quantified using fluorometry with an Oroboros fluorespirometer. Subsequently, select ROS emission sites in the mitochondrial complexes were isolated using inhibitors, and the ROS emission of each site was measured using the Amplex UltraRed-horseradish peroxidase (AUR-HRP) system. The compound 3,5-dimethyladamantane-1-acetic acid was equally potent in causing ROS emission in State 3 and State 4 ROS sites. The baseline (no NAFC) proportion of ROS emission by site was II_F_ > I_Q_ > III_Qo_ > I_F_. The 3,5-dimethyladamantane-1-acetic acid compound increased ROS emission in a dose-dependent manner at II_F_ with an EC_50_ of 0.2 mM, which was not significantly different than the State 3 and 4 Oroboros response. In contrast, there was no consistent concentration-effect response at the other three ROS sites (I_Q_, III_Qo_, and I_F_). Malonate, an inhibitor of succinate dehydrogenase, eliminated ROS production in Oroboros experiments. These findings identify site II_F_ as the predominant source of NAFC-stimulated ROS and provide mechanistic insight into how adamantane-type NAFCs impair mitochondrial redox balance in fishes.

## 1. Introduction

Most reactive oxygen species (ROS) are produced by mitochondria, with the highest concentrations observed in the heart, liver, and swim bladder during rest and in red muscle during swimming [1,2]. This phenomenon occurs as a byproduct of the single-electron reduction of oxygen within the electron transport chain (ETC) of mitochondria, leading to the generation of the superoxide anion (O_2_^•−^), which subsequently transforms into hydrogen peroxide (H_2_O_2_).

Most of this H_2_O_2_ is metabolized by catalase; however, excess H_2_O_2_ can produce the highly reactive hydroxyl radical (^•^OH) [3]. The damaging effects of ROS have posed serious problems for life forms ever since aerobic metabolism evolved [4]. ROS plays a dual role in cell physiology, acting as crucial signalling molecules in several biological processes and as a primary cause of cellular damage [4]. ROS steady-state concentration represents an equilibrium between production and removal; disruption of this balance, known as oxidative stress, results in elevated levels of ROS and damage to cellular components, leading either to signalling (eustress) or to cellular damage (distress) [5]. Oxidative stress plays a central role in the mechanism of toxicity, as ROS can damage DNA, lipids, and proteins, which ultimately impair cell function, triggering various cell death modalities, including apoptosis, necrosis, and others [6]. Therefore, ROS can be a potential key mediator in chemical, environmental, and physiological toxicity [7].

Organic acids, such as fatty acids, resin acids from pulp and paper wastewater [8], and more recently polyfluoroalkyl compounds have shown the ability to cause toxicity through energetic or mitochondrial mechanisms [9]. Naphthenic acid fraction compounds (NAFCs) are a complex family of carboxylic acids found in oil sand process-affected water (OSPW) [10]. NAFCs are thought to be the primary acutely lethal component found in Canadian oil sands [11]. OSPW exposure in rainbow trout (*Oncorhynchus mykiss*) caused behavioural disturbances [12], altered immune function [13], and disrupted mitochondrial function in red muscle tissue, which impairs swimming performance [14].

Mechanistic studies using NAFCs have found that when mitochondria from rainbow trout liver were exposed to a different NAFC mixture extracted from OSPW, mitochondrial oxidative phosphorylation (OXPHOS) was reduced in state 3 and state 4 respiration. In mitochondrial respiration, the addition of adenosine diphosphate (ADP) initiates State 3, where adenosine triphosphate (ATP) is actively generated. Once the added ADP is consumed, mitochondria transition to State 4, during which no ATP is produced. This reduction was measured as a drop in oxygen consumption, reduced mitochondrial membrane potential (Δψ_m_) and concomitant ROS (H_2_O_2_) emission increases [15]. Nearly identical effects were found using a commercially available adamantane carboxylic acid, 3,5-dimethyladamantane-1-carboxylic acid [16]. Further exploration of this mechanism with a second, more potent, adamantane carboxylic acid, 3,5-dimethyladamantane-1-acetic acid, found that mitochondrial complex protein activity was selectively inhibited with the sensitivity of complex IV (CIV) ≥ complex I (CI) > complex III (CIII) > complex II (CII) over nearly an order of magnitude difference in dose-effect [17]. Identifying the specific mitochondrial sites of ROS generation is critical for understanding the mechanism of NAFC toxicity and for developing targeted mitigation strategies.

The exact localization of mitochondrial sources of ROS remains controversial. Some studies suggest CI is the primary contributor, while others identify CIII or dehydrogenase-associated sites, indicating a lack of consensus [18,19]. The mitochondrial complex location of ROS production in response to NAFCs is unknown. Twelve mitochondrial sites where O_2_^•−^ and H_2_O_2_ are generated within the citric acid cycle and the ETC associated with OXPHOS have been identified [20,21]. These sites are grouped according to the redox potential they operate at into nicotinamide adenine dinucleotide (reduced form)/nicotinamide adenine dinucleotide (oxidized form) (NADH/NAD^+^) and ubiquinol/ubiquinone (QH_2_/Q) isopotential pools [22]. The sites associated with NADH/NAD^+^ isopotential pool include 2-oxoglutarate dehydrogenase (flavoprotein) (O_F_), pyruvate dehydrogenase (P_F_), branched-chain 2-oxoacid dehydrogenase (flavoprotein) (B_F_), and 2-oxoadipate dehydrogenase (flavoprotein) (A_F_) within the 2-oxoacid dehydrogenase complexes; flavin site (I_F_) and ubiquinone site (I_Q_) in CI. The QH_2_/Q isopotential pool includes the outer quinol-binding site (III_Qo_) in CIII; and flavin site in CII (II_F_), and additional sites located on Q-dependent dehydrogenases [22].

The goal of this study was to determine whether H_2_O_2_ emission is stimulated by 3,5-dimethyladamantane-1-acetic acid at specific sites within the mitochondrial ETC under different bioenergetic states. Based on previous evidence that NAFCs impair mitochondrial complexes [17], it was hypothesized that exposure to this compound would alter ROS emission from one or more electron transport sites. Determining the site specificity of NAFC-induced ROS is important for understanding how NAFCs disrupt mitochondrial function in fishes. Initially, the emission of ROS during States 3 and 4 respiration was quantified in isolated rainbow trout liver mitochondria using a high-resolution respirometer. Subsequently, ROS-producing sites in mitochondrial enzymes were isolated using inhibitors, and their ROS emission capacities were measured, followed by a confirmatory experiment using an inhibitor in an Oroboros experiment.

## 2. Materials and Methods

### 2.1. Experimental Design

The influence of a naphthenic acid (NA), 3,5-dimethyladamantane-1-acetic acid (Sigma Aldrich, Oakville, ON, Canada; CAS number 14202-14-3), on the H_2_O_2_ metabolism and ROS production of intact rainbow trout mitochondria was examined using fluorometry. The Amplex UltraRed (AUR) and horseradish peroxidase (HRP) were used to study the impact of 3,5-dimethyladamantane-1-acetic acid on site-specific H_2_O_2_ production within the mitochondrial ETC system. AUR indirectly measures H_2_O_2_ by reacting with it in an HRP-catalyzed reaction to produce the fluorescent compound resorufin. The mitochondria were treated with appropriate pharmacological agents, ETC inhibitors, to isolate ROS production sites chemically. The contribution of H_2_O_2_ production from the sites I_F_, I_Q_, II_F_, and III_Qo_ following exposure to 3,5-dimethyladamantane-1-acetic acid was measured in response to 3,5-dimethyladamantane-1-acetic acid using a microplate fluorometric assay. The effect of 3,5-dimethyladamantane-1-acetic acid on site-specific ROS emission was estimated by exposing mitochondria with the specific sites sequestered to 7 concentrations of 3,5-dimethyladamantane-1-acetic acid and the carrier ethanol as a control. Mitochondria isolated from individual fish were used as biological replicates.

### 2.2. Chemicals

All chemicals and reagents used to make buffers, mitochondrial protein quantification, assay reagents, substrates, and inhibitors used for the study were sourced from Sigma-Aldrich (Burlington, MA, USA) except AUR and HRP, which were obtained from ThermoFisher (Waltham, MA, USA).

### 2.3. Animals, Liver Mitochondrial Isolation, and Buffers

Rainbow trout were housed at the aquatic facility at the Atlantic Veterinary College of the University of Prince Edward Island. *Female juvenile* fish (400–600 g) were obtained from Ocean Farms (Brookvale, PEI, Canada) and held in an aerated flow-through well-water 250 L tank with a flow rate of 20 L/min maintained at 11 ± 1 °C. The well water had a pH of 7.7, with a hardness of 310.7 mg/L and an alkalinity of 153 mg/L. The fish were provided with a daily diet consisting of commercial trout feed (Corey Feed Mills, Fredericton, NB, Canada) at a rate of 1% of their body weight. The University of Prince Edward Island Animal Care Committee approved the research and all experimental methods in compliance with the Canadian Council on Animal Care (protocol #21-036).

Mitochondria were isolated using standard differential centrifugation. Fish were euthanized by stunning followed by cervical transection, and liver tissue (~2.3 g) was excised and rinsed in mitochondrial isolation buffer (MIB). The MIB contained 250 mM sucrose, 10 mM Tris hydrochloride (Tris-HCl), 10 mM potassium phosphate (KH_2_PO_4_), 0.5 mM ethylene glycol bis (-aminoethyl ether)-N, N′-tetraacetic acid (EGTA), 1% fatty acid-free bovine serum albumin (BSA), and 2 μg/mL aprotinin. The liver samples were then cut into small pieces and blended in a 1:3 ratio of tissue to MIB solution using a 10-mL Potter–Elvehjem homogenizer (Cole Parmer, Montreal, QC, Canada). This was achieved by making four passes with a Teflon pestle attached to a hand-held drill (Mastercraft, MAS 2BB, Toronto, ON, Canada) at a speed of 200 rpm. The homogenate obtained was thereafter subjected to centrifugation at a speed of 800× *g* for a duration of 15 min at a temperature of 4 °C. The supernatant was centrifuged at 13,000× *g* for 10 min, followed by washing the mitochondrial pellet twice, resuspending it in MIB, and centrifuging at 11,000× *g* for 10 min at 4 °C. The mitochondrial pellet was weighed and resuspended in a mitochondrial respiration buffer (MRB) solution consisting of 10 mM Tris-HCl, 25 mM KH_2_PO_4_, 100 mM potassium chloride, 1 mg/mL BSA, and 2 μg/mL aprotinin, at a ratio of 1:3 (weight to volume). Both MIB and MRB had a pH of 7.3 in parallel with the fish’s intracellular pH range. The protein concentration (1 mg/mL) of the mitochondrial suspension was determined using the Bradford technique (1976) using BSA as the reference standard.

### 2.4. Measurement of Mitochondrial ROS Emission

An Oxygraph-2k FluoRespirometer (Oroboros Instruments, Innsbruck, Austria) was used to measure ROS generation. Both H_2_O_2_ concentration and H_2_O_2_ flux were measured fluorometrically using the fluorophore AUR and an H_2_O_2_ sensor. At the beginning of each experiment, H_2_O_2_ was calibrated by initially filling the 2 mL chambers with MRB [17]. Then the constituents of the H_2_O_2_ detection system, AUR (25 μM and HRP (0.5 U/mL), were added, and an H_2_O_2_ calibration curve (0–0.45 μM/mL) was then generated to convert fluorescence intensity into H_2_O_2_ concentration. Following calibration, real-time measurements of ROS production were obtained (pmol/mL/s), which were subsequently normalized to mitochondrial protein content during data analysis.

The study employed a paired-chamber design. Each chamber contained isolated mitochondria (1 mg protein/mL) supplied with glutamate and malate (5 mM) as substrates to energize the mitochondria. Following the injection of a 1 mM ADP bolus, the mitochondria entered State 3 respiration, characterized by active ATP production. Once all ADP was consumed and ATP synthesis ceased, the mitochondria transitioned to State 4 respiration. When steady State 3 or 4 was reached, seven serial doses of 3,5-dimethyladamantane-1-acetic acid were administered in the experimental chamber, while ethanol was added in parallel as the carrier control. Dose-effect curves consisting of concentrations ranging from 0 to 14 mM (0 to 3125 mg/L) were obtained during State 3 respiration and then again during State 4 respiration to investigate the effects of 3,5-dimethyladamantane-1-acetic acid on the production of H_2_O_2_ in two distinct mitochondrial energetic States, State 3 and State 4. Each endpoint for States 3 and 4 (H_2_O_2_ production) was subjected to a minimum of 12 replicates, with State 3 and State 4 assessed in separate chamber runs using mitochondrial preparations derived from distinct fish. Each run in the paired chambers represented technical replicates for a single fish (biological replicate). EC_50_ values for State 3 and State 4 ROS emission were calculated from these concentration–effect curves.

### 2.5. Measurement of Site-Specific ROS Production in Isolated Mitochondria

In NA-exposed liver mitochondria, four distinct sites of H_2_O_2_ production, I_F_, I_Q_, II_F_, and III_Qo_, within the ETC and related enzymes were examined using site-specific inhibitors. Mitochondrial H_2_O_2_ production was measured with the standard assay in 200 μL microplate wells containing horseradish HRP, substrates, and inhibitors, across a concentration range of 0–14 mM (0–3125 mg/L) 3,5-dimethyladamantane-1-acetic acid, with ethanol used as the carrier control.

These were added one at a time to each microplate well, followed by mitochondria suspension (1 mg protein per ml assay concentration) and AUR. In this assay, the mitochondria produce H_2_O_2_, which catalyzes the conversion of the non-fluorescent AUR into a fluorescent product, which emits light at a wavelength of 590 nm when excited at a wavelength of 530 nm. The fluorescence data were obtained for a duration of 30 min using a microplate fluorescence reader (Synergy^TM^ HT BioTek, Winooski, VT, USA). Fluorescence intensities were converted to H_2_O_2_ concentration using standard curves of H_2_O_2_ generated simultaneously, while also accounting for background fluorescence. Each concentration was measured using duplicate technical wells per fish. Mitochondria from 9 individual fish were used as biological replicates across all site-specific assays. For assays in which a concentration–effect relationship was observed, EC_50_ values were derived from the corresponding concentration–effect curves.

### 2.6. Measurement of the Flavin Site in Complex I (I_F_) ROS Production

Site I_F_ H_2_O_2_ emission was quantified with isolated liver mitochondria energized with 5 mM of malate as substrate (to reduce NAD^+^ to NADH) and a classical Q-site inhibitor of CI, which is 4 μM of rotenone to inhibit reoxidation and block exit of electrons from CI by the Q-pool, to reduce the flavin mononucleotide site fully, and to collapse protonmotive force. To reduce the amount of forward electron flow at the 2-oxoglutarate dehydrogenase complex (OGDH), which contributes to the production of H_2_O_2_ at site I_F_, 1.5 mM aspartate was added to eliminate the naturally occurring 2-oxoglutarate through transamination. Additionally, 2.5 mM ATP was added to decrease carbon flow at different stages of the Krebs cycle, specifically targeting succinate thiokinase. To calculate the contribution of site I_F_ in ROS production, the rates of H_2_O_2_ production from wells containing malate and rotenone were subtracted from those containing malate, rotenone, ATP, and aspartate.

### 2.7. Measurement of the Ubiquinone Binding Site in Complex I (I_Q_) ROS Production

Site I_Q_ H_2_O_2_ emission was quantified with isolated liver mitochondria energized with 5 mM of succinate as substrate to reduce Q to QH_2_ and generate protonmotive force to drive reverse electron transport and 4 μM of rotenone as a specific inhibitor to block the Q-reducing site of CI. To calculate the contribution of site I_Q_ in ROS production, the rates of H_2_O_2_ production from wells containing succinate and rotenone were subtracted from those containing succinate only.

### 2.8. Measurement of the Flavin Site in Complex II (II_F_) ROS Production

Site II_F_ H_2_O_2_ emission was quantified in isolated liver mitochondria energized with 0.1 mM succinate, which supports ROS production from site II_F_ in the forward reaction. To eliminate ROS contributions from CI (site I_Q_) and CIII (site III_Qo_), 4 μM rotenone and myxothiazol were used, respectively. Finally, ROS emission from site II_F_ was defined as the portion sensitive to 5 mM malonate, a specific inhibitor of site II_F_, eliminating emission by both the forward and reverse reactions. The site II_F_ ROS emission was calculated as the difference between the rates obtained with and without malonate, that is, with succinate, rotenone, and myxothiazol minus succinate, rotenone, myxothiazol, and malonate. For site II_F_, where a clear concentration–effect relationship was present, EC_50_ values were calculated from the resulting concentration–effect curves.

#### Measurement of Mitochondrial ROS Emission at Site II_F_ Using Respirometer

Mitochondria were energized with 0.1 mM succinate in the Oroboros respirometer. The paired-chamber design was used: one chamber contained mitochondria with ethanol as the carrier control, and the other chamber contained mitochondria with 3,5-dimethyladamantane-1-acetic acid at its EC_50_ dose of 0.2 mM. In addition to the carrier control, a separate condition was tested in which energized mitochondria were exposed to 3,5-dimethyladamantane-1-acetic acid alone to assess ROS production in the absence of site-specific inhibition. To specifically assess ROS from site II_F_, the emission sensitive to the site II_F_-specific inhibitor, malonate (5 mM), was determined while all other mitochondrial complexes and sites remained uninhibited. As inhibitors themselves can artificially induce ROS production, examining ROS emission with and without malonate at CII provides a verification of whether site II_F_ is a significant contributor to NAFC-induced ROS. ROS production was measured as described in Section 2.4.

### 2.9. Measurement of the Outer Quinol-Binding Site in Complex III (III_Qo_) ROS Production

Site III_Qo_ H_2_O_2_ emission was quantified with isolated liver mitochondria energized with 5 mM of succinate as substrate (to reduce Q to QH_2_) and generate protonmotive force to drive forward electron transport, 4 μM of rotenone to prevent CI superoxide production at site I_Q_, 2 μM of antimycin A (AA) as an inner Q binding site of CIII (site III_Qi_) site inhibitor to block the exit of electrons from CIII and collapse protonmotive force, and 4 μM of myxothiazol as a specific inhibitor of site III_Qo_ to inhibit the contribution of ROS from site III_Qo_ in CIII. To calculate the contribution of site III_Qo_ in ROS production, the rates of H_2_O_2_ production from wells containing succinate, rotenone, AA, and myxothiazol were subtracted from those containing succinate, rotenone, and AA only. All site-specific ROS measurements were conducted using mitochondria from individual fish as biological replicates, with duplicate technical wells per condition.

### 2.10. Statistics

Half maximal effective concentration (EC_50_) values were calculated using a four-parameter logistic concentration-effect equation of the form (bottom + (top-bottom))/(1 + 10(log (EC_50_)-log (Concentration)) × Hillslope) to evaluate each replicate fish concentration-effect curve for ROS emission endpoints using nonlinear regression to estimate EC_50_ values in Statistica v 13.5 (Tibco Software Inc., Palo Alto, CA, USA). The top and bottom represented the maximal and minimal responses, respectively. Concentration is the exposure amount; EC_50_ is the median effective concentration; and Hillslope is a constant representing the steepness of the slope. A quasi-Newton estimation method with a standard loss function ((observed-predicted)^2^) and a convergence criterion of 0.0001 for the loss function was used for nonlinear curve fitting. For visualization of concentration-effect curves, a composite curve was generated using data from all replicated experiments expressed as a percentage of the top value from the curve fit. However, all statistics were conducted on the EC_50_ values generated from concentration-effect curves generated for each replicate fish. Therefore, statistical analyses were performed on biological replicates (individual fish).

All statistics were conducted using two-way analysis of variance (ANOVA), where there were only two treatments, or by ANOVA followed by Tukey’s post hoc tests for multiple comparisons. The assumption of normality was tested using normal probability plots, and the assumption of homogeneity of variances was tested using the Brown-Forsythe test. Logarithmic transformations were applied where there were deviations from the assumptions of parametric statistics. All responses were expressed relative to the carrier controls. Experiment-wise alpha was set at 0.05 for all analyses. Statistica v 13.5 was used for all statistical analyses. Error bars represent the SEM unless otherwise noted.

### 2.11. Data Availability

All data generated or analyzed in this study are included in this article, and additional raw data will be provided upon request. There are no restrictions on the availability of materials or data.

## 3. Results

### 3.1. Concentration-Dependent ROS Emission in the Oroboros Respirometer

**Figure 1:**
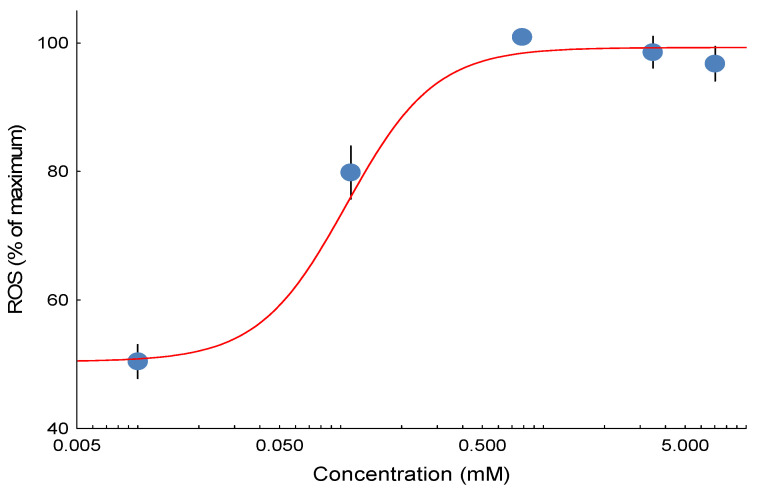
Representative concentration-effect curve for 3,5-dimethyladamantane-1-acetic acid-induced ROS emission. All individual fish are scaled to the fitted curve maximum (100%) in order to visualize the relative response over all experiments. Data represent biological replicates (n = 12 fish), with paired chambers serving as technical replicates. Error bars represent the standard error of the mean (SEM).

The 3,5-dimethyladamantane-1-acetic acid model NAFC used in the current study induced ROS emission in isolated mitochondria in a concentration-dependent pattern as measured in the Oroboros respirometer in state 3 (Figure 1). While the overall shape of the concentration effect for ROS is typically Gaussian curves were fitted to only the lower concentration portion of the response to estimate the median potency for the stimulation of ROS. At the highest concentrations, ROS emission ceased, likely reflecting the complete loss of mitochondrial function.

### 3.2. Comparison of State 3 and State 4 Potency and Relative Potency to Other NAFCs

**Figure 2:**
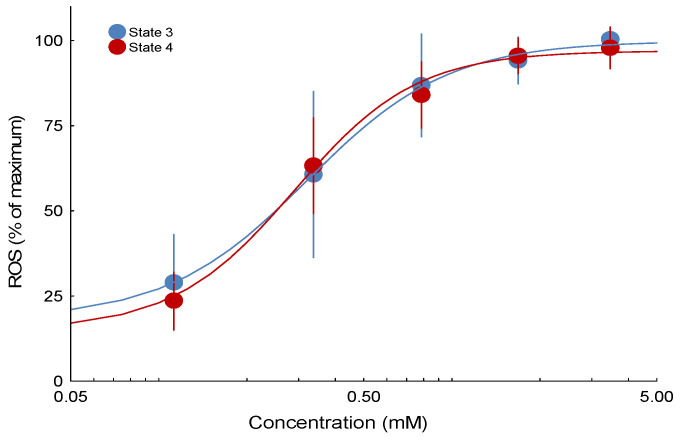
Concentration-effect curves for mitochondrial ROS production in States 3 and 4 in response to the NAFC, 3,5-dimethyladamantane-1-acetic acid. Responses are scaled to maximum values based on the curve fit for each fish (100%) for comparison purposes. Error bars indicate the SEM. Paired chambers served as technical replicates for each fish. The number of fish (n) ranges between 9 and 10 fish for each point.

There was no statistically significant difference in the potency of the compound to stimulate a mitochondrial H_2_O_2_ emission rate in State 3 compared with State 4 (Figure 2). The compound tested herein was approximately 10-fold more potent at ROS generation than the previously tested adamantane carboxylic acid, 3,5-dimethyladamantane-1-carboxylic acid (Table 1). The 3,5-dimethyladamantane-1-acetic acid tested herein was only modestly more potent than an extracted and purified NAFC mixture derived from oil sand process affected waters (Table 1).

### 3.3. Baseline Site-Specific ROS Emission and Effect of Ethanol

**Figure 3:**
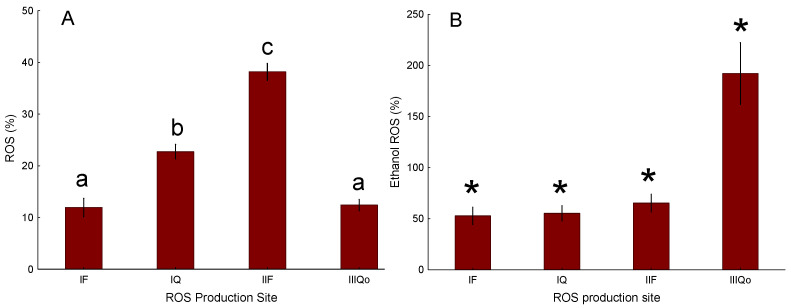
Mean ROS emission for (**A**) untreated mitochondria, where ROS from each site is expressed as a percentage of the total cumulative ROS across all sites in untreated mitochondria; (**B**) mitochondria treated with ethanol (carrier control), where ROS at each site is expressed as a percentage of the corresponding untreated mitochondria at that site. Error bars represent the SEM. For panel (**A**), the means not sharing a letter are significantly different; for panel (**B**), the means with asterisks are significantly different from the corresponding untreated control. Data were obtained using mitochondria from 9 individual fish (biological replicates), with duplicate technical wells per condition.

The relative response of untreated mitochondria using the ROS plate reader assay with chemically suppressed ROS sites revealed a statistically significant differential emission of ROS at the various sites in baseline conditions (Figure 3A). The order of relative ROS response was II_F_ > I_Q_ > III_Qo_ > I_F_. The range in response of ROS emission between sites was approximately fourfold. The ethanol carrier control had a differential effect on those baseline ROS emission values (Figure 3B). ROS emission at site III_Qo_ was almost doubled. In contrast, ROS emission at sites I_F_, I_Q_, and II_F_ were reduced by approximately half. Due to the ethanol response, all subsequent analyses were evaluated relative to the ethanol control.

### 3.4. Site-Specific ROS Emission Induced by 3,5-Dimethyladamantane-1-Acetic Acid

**Figure 4:**
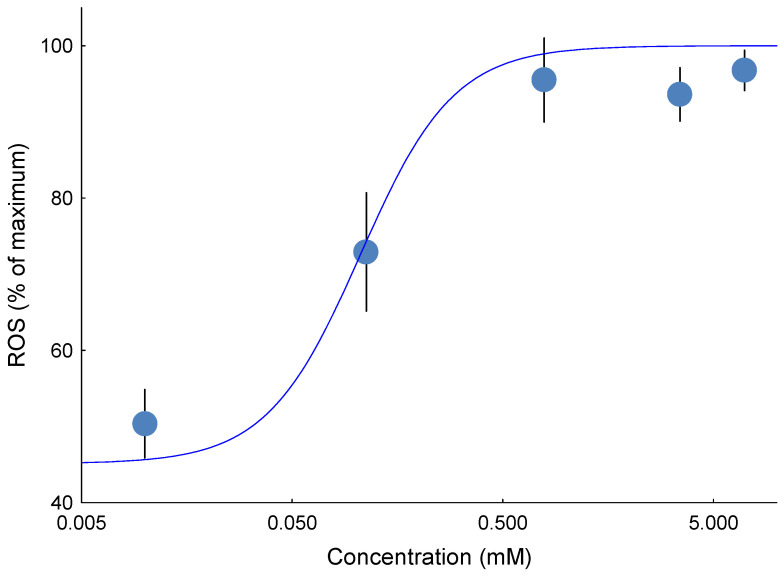
Representative concentration–effect curves showing stimulation of H_2_O_2_ emission by 3,5-dimethyladamantane-1-acetic acid in succinate-energized mitochondria (0.1 mM). Emission from site II_F_ of the QH_2_/Q pool was enhanced in a concentration-dependent manner compared with other sites. Data were obtained using mitochondria from 9 individual fish (biological replicates), with duplicate technical wells per concentration. Error bars represent the SEM.

**Figure 5:**
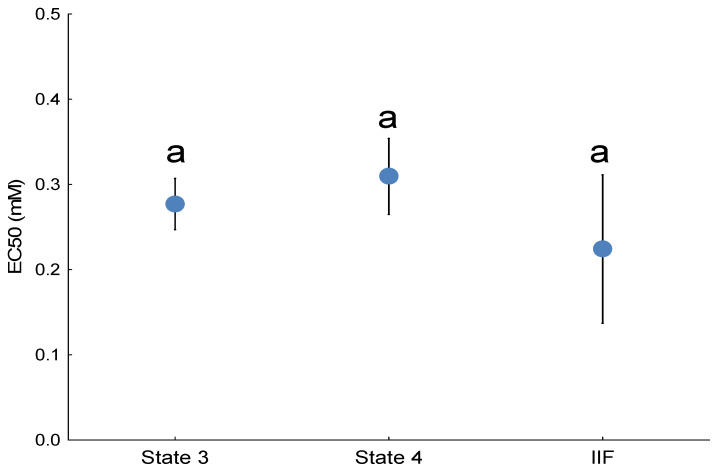
Mean EC_50_ for 3,5-dimethyladamantane-1-acetic acid-induced ROS emission in State 3 and State 4 respiration as compared to 3,5-dimethyladamantane-1-acetic acid-induced ROS emission of chemically isolated site II_F_. Error bars are SEM. Means with a common subscript are not significantly different. State 3 and State 4 EC_50_ values were derived from mitochondria of 12 individual fish, with paired chambers serving as technical replicates. The site II_F_ EC_50_ value was derived from mitochondria of 9 individual fish, with duplicate technical wells per concentration. The letter ‘a’ denotes that there were no significant differences among the groups.

The effects of NAFC 3,5-dimethyladamantane-1-acetic acid on mitochondrial H_2_O_2_ emission depended on the substrate used and the redox site being measured. For mitochondria oxidizing a sub-saturating concentration (0.1 mM) of succinate, the 3,5-dimethyladamantane-1-acetic acid (Figure 4) influenced the H_2_O_2_ emission rate from site II_F_ in a concentration-dependent manner. Specifically, NAFC 3,5-dimethyladamantane-1-acetic acid concentration-dependently stimulated H_2_O_2_ emission from the site II_F_ (Figure 4), and no stimulation was detected at the other analyzed sites I_F_, I_Q_, and III_Qo_. The II_F_ EC_50_ was not statistically different than those estimated for State 3 and State 4 respiration using the Oroboros (Figure 5)*,* and this implies that all the ROS emission stimulated using the Oroboros technique originated from site II_F_.

### 3.5. Confirmation of Induced Site II_F_ ROS Emission in Intact ETC (Oroboros Assay)

**Figure 6:**
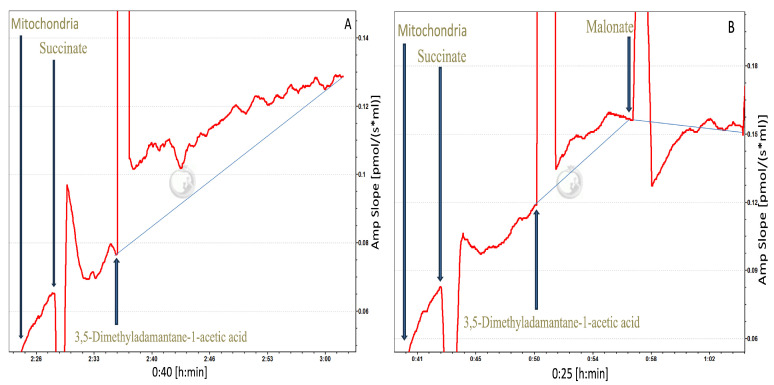
Fluorometric detection of mitochondrial ROS production at site II_F_ following exposure to 3,5-dimethyladamantane-1-acetic acid (0.2 mM; EC_50_) under succinate-energized conditions (0.1 mM). A blue guideline line is drawn to illustrate the overall change in ROS slope over time. ROS increased after NAFC application, and the upward trend is indicated by the rising blue line. The subsequent addition of 5 mM malonate resulted in a cessation of ROS emission, depicted by the downward shift in the blue line, confirming site II_F_ as the primary source of NAFC-induced ROS. (**A**) shows mitochondria energized with succinate and exposed to 3,5-dimethyladamantane-1-acetic acid, where ROS production progressively increased over time; (**B**) shows the same treatment followed by malonate addition, demonstrating inhibitor-sensitive suppression of ROS. These traces represent three independent biological replicates (three individual fish), with paired chambers serving as technical replicates for each experiment.

To confirm that the ROS increase was directly induced by NAFC at site II_F_ in an intact ETC, and not influenced by artifacts from isolating ROS sites with multiple inhibitors, additional Oroboros experiments were performed. Mitochondria were energized with 0.1 mM succinate. NAFC 3,5-dimethyladamantane-1-acetic acid was applied at its EC_50_ dose (0.2 mM) to stimulate ROS, and the site II_F_-specific inhibitor malonate (5 mM) was then added, which reduced ROS (Figure 6). All other mitochondrial complexes and sites remained fully functional, allowing the intact ETC to operate simultaneously. The site II_F_–specific contribution of NAFC-induced ROS was quantified by comparing changes *in* the ROS increase after NAFC 3,5-dimethyladamantane-1-acetic acid addition with and without subsequent malonate inhibition. Across three independent mitochondrial preparations, the percent II_F_-specific ROS was 63.5%, 76.5%, and 65.4% for fish 1, 2, and 3, respectively (mean ± SEM: 68.46% ± 4.05%). These results indicate that a substantial portion of NAFC-stimulated ROS originates specifically from site II_F_. These results confirm that NAFC stimulates ROS specifically at site II_F_ under physiologically intact conditions, indicating that the ROS observed in the plate reader assay reflects a genuine effect of the toxicant rather than an artifact of downstream inhibition.

## 4. Discussion

These findings indicate that 3,5-dimethyladamantane-1-acetic acid selectively stimulates ROS at specific sites within the ETC, particularly site II_F_. Exposure of isolated rainbow trout mitochondria to the model NAFC 3,5-dimethyladamantane-1-acetic acid showed equivalent potency for inducing ROS emission in both State 3 and State 4 respiration. Untreated mitochondria show that the largest proportion of ROS originates from site II_F_. Isolation of ROS sites showed that only site II_F_ showed a quantifiable concentration-effect that was equipotent to the ROS emission effect observed in State 3 and State 4. The NAFC 3,5-dimethyladamantane-1-acetic acid was an order of magnitude more potent as a ROS inducer than a previously tested and structurally similar compound, 3,5-dimethyladamantane-1-carboxylic acid, and was moderately more potent than a mixture of NAFCs extracted from OSPW.

The mechanism by which ROS is generated in mitochondria in response to NAFCs is through inhibition of the ETS downstream, causing a backup of electrons and increased leakage from sites upstream of the point of inhibition. In contrast, sites beyond the inhibition point become oxidized and therefore produce little ROS. At lower NAFC concentrations, additional impairment of ROS scavenging systems may further contribute to elevated ROS emission, as observed at site II_F_ in the present study. At high NA concentrations, the associated reduction in electron flow and Δψ_m_ would diminish ROS production, consistent with the complete loss of mitochondrial function observed. Both NAFCs extracted from OSPWs and the adamantane carboxylic acid 3,5-dimethyladamantane-1-carboxylic acid impair rainbow trout mitochondrial respiration, thereby reducing Δψ_m_ [15,16]. Any direct disruption of the complexes in the ETC by NAFCs would diminish electron flow, heightening the likelihood of leakage and consequently elevating H_2_O_2_ emission. Experiments to specifically isolate mitochondrial sites at which respiration is impaired were conducted by examining the activity of chemically isolated mitochondrial complexes in response to exposure to the same NAFC used in our previous study [17]. All mitochondrial complexes were impaired by 3,5-dimethyladamantane-1-acetic acid. Still, there was an order of magnitude difference in potency, with the potency order (lower to higher half maximal inhibitory concentration (IC_50_)) being CIV ≥ CI > CIII > CII. Previous studies have shown that respiratory CII, as part of the Krebs cycle, oxidizes succinate to fumarate and reduces Q in the ETC; therefore, site II_F_ can generate high rates of H_2_O_2_ when electrons accumulate due to inhibition of CI and CIII in the presence of succinate [23]. In the case of 3,5-dimethyladamantane-1-acetic acid examined herein, inhibition of complex activity both upstream and downstream of CII would be expected to increase ROS emission from site II_F_.

The concentration–effect relationship indicates that ROS production arises from upstream and, particularly, downstream inhibition of complex activity. In this study, 3,5-dimethyladamantane-1-acetic acid induced ROS emission with an EC_50_ of 0.2–0.3 mM, whereas overall respiration was inhibited at an IC_50_ of 0.8–1 mM and CIV activity at 1.2 mM [17]. Thus, the median ROS emission effect occurred at approximately five-fold lower concentrations than those required to inhibit respiration. A similar relationship between ROS and respiration has been reported for oil sands–extracted NAFCs [15]. This pattern can be explained by the fact that ROS production requires a partially functional ETC; once respiration is severely compromised, mitochondria can no longer produce ROS. Accordingly, ROS emission is most pronounced during the early stages of respiratory inhibition, corresponding to the ten percent effective concentration for respiration [15], and declines at higher concentrations, giving rise to the typical Gaussian-shaped ROS response curve.

The relative abundance of mitochondrial substrates influences ROS production. CII is a recognized source of ROS during succinate oxidation [24]. This theory explains why we observed ROS stimulation at II_F_ due to NAFC during succinate oxidation. In contrast, electrons from malate oxidation reduce NAD^+^ to NADH, which delivers them to CI, which in turn reduces Q to QH_2_. QH_2_ then delivers electrons to CIII; under these conditions, respiratory inhibition or NAFC-induced uncoupling can reduce the production of ROS at site III_Qo_.

Collectively, these findings highlight that NAFC-induced ROS emission is substrate-dependent and site-specific, with succinate oxidation at site II_F_ being particularly susceptible. These findings indicate that within the QH_2_/Q pool, site II_F_ responds differently to NAFC exposure than site III_Qo_. Specifically, H_2_O_2_ emission at site II_F_ was increased in oxidized mitochondria exposed to 3,5-dimethyladamantane-1-acetic acid, whereas site III_Qo_ was unresponsive to the model NAFC when mitochondria were energized with high concentrations of succinate as the substrate. This suggests that NAFCs can enhance electron leakage at site II_F_.

The idea of a single site being responsible for most of the toxicant-induced and baseline ROS emission may be species- or tissue-specific. In rat skeletal muscle mitochondria, CII produces high ROS levels in both forward and reverse reactions, with site II_F_ being the sole source [23]. Another study, again in rodent skeletal muscle mitochondria, showed that site I_Q_ and site II_F_ dominate H_2_O_2_ production at rest, whereas site I_F_ dominates under aerobic exercise conditions [25]. Moreover, in primary astrocytes maintained under hypoxic conditions, site I_Q_ plays a crucial role in baseline ROS generation, indicating that a singular mitochondrial site may mostly influence baseline ROS levels [21]. It was observed that in mice lacking glutaredoxin-2 (GRX2), the major mitochondrial ROS sites differed between tissues [26]. In liver mitochondria from GRX2-deficient mice, OGDH and CIII were the main emitters, whereas in cardiac mitochondria from the same animals, CI and CIII dominated. In addition to findings in mammalian tissues, studies on rainbow trout heart mitochondria have shown that baseline H_2_O_2_ emission follows a hierarchical pattern among mitochondrial sites, III_Qo_ > II_F_ ≥ I_F_ > I_Q_, demonstrating that different sites contribute variably to ROS production even under unstressed conditions [27]. On the other hand, the overall H_2_O_2_ emission capacities of the relevant sites in fish liver mitochondria that oxidize succinate were II_F_ > III_Qo_ > I_Q_ [28]. Our results are consistent with the idea that the predominance of a given ROS-producing site is species- or tissue-specific and indicate that the highest H_2_O_2_ emission potential in untreated energized mitochondria can be found at sites II_F_, I_Q_, III_Qo_, or I_F_, depending on the oxidized substrate.

The use of carrier substances such as ethanol in testing hydrophobic substances can be problematic in the examination of mitochondrial energetics. In the present study, ethanol influenced ROS production from all the sites assessed in untreated energized mitochondria. Our findings are consistent with other recent studies showing that ethanol can increase or reduce ROS production in the mitochondria. Interestingly, previous research highlighted how ethanol increases ROS levels in the mitochondria, which was linked to its metabolism through oxidative processes, modifications to the mitochondrial ETC, or both [29]. Thus, our study supports the idea that even low ethanol concentrations can affect mitochondrial redox status in vitro.

The unavailability of pure NAFCs with which to conduct toxicological studies has hampered the assessment of the risk of these environmentally significant compounds. Herein, it is shown that the adamantane carboxylic acid 3,5-dimethyladamantane-1-acetic acid has promise as a model compound due to both its ability to induce ROS at a similar concentration to oil sands extracted mixtures [15]. Multiple sources of OSPW have been found to contain adamantane acids, including the compound at hand [30,31]. Tricyclic diamondoid or adamantane NAs are likely biotransformation products of adamantane hydrocarbons and thus relatively ubiquitous [30]. Researchers have found that older tailings samples have more adamantane NAFCs, suggesting a recalcitrance to environmental breakdown [30]. The relative strength of adamantane compounds as stand-ins for oil sands NAFCs is in part due to their economical commercial accessibility in quantities suitable for experimentation, as well as their ability to mimic the mechanistic mitochondrial responses of OSPW-NAFCs.

ROS are signalling molecules in cellular pathways; however, excessive production or accumulation of ROS can result in redox imbalances and biomolecular damage. Many environmental stressors harm aquatic organisms by triggering excessive ROS production that overwhelms cellular defences and causes widespread oxidative damage. However, understanding how environmental stressors affect aquatic organisms’ mitochondria and change the generation of mitochondrial ROS is still lacking. Our study examined how exposure to a model NAFC affected rainbow trout liver mitochondrial H_2_O_2_ emission. This, in combination with other studies, shows that 3,5-dimethyladamantane-1-acetic acid, akin to other NAFCs [16], inhibited ETS, shown by a decline in State 3 and 4 respiration [17] and elevated H_2_O_2_ production. Whether adamantane acetic acids can adequately serve as substitutes for OSPW-NAs in toxicological assessments warrants further investigation. The link between ROS emission in response to NAFCs exposure and cell death, and what specific modalities are involved, needs to be investigated. Gaining more insight into the mechanisms of toxicity of adamantane-type NAs will enhance our understanding of the biological consequences and environmental risk posed by OSPW containing these acids. The results of the present study reinforce those of other studies showing that very low concentrations of NAFC are toxic, highlighting the importance of stricter thresholds for NAFC exposure to protect aquatic health.

## Figures and Tables

**Table 1:** Mean (SEM, n) EC_50_ values (mM) of 3,5-dimethyladamantane-1-acetic acid with respect to ROS production endpoints compared with the previously analyzed 3,5-dimethyladamantane-1-carboxylic acid [16].

Parameter (mM)	3,5-Dimethyladamantane-1-Acetic Acid	3,5-Dimethyladamantane-1-Carboxylic Acid ^a^	Extracted NAFC ^a^
State 3 H_2_O_2_ emission	0.27 (0.03, 9)	2.57 (1.39, 3)	0.88 (0.22, 4)
State 4 H_2_O_2_ emission	0.31(0.04, 10)	2.68 (1.09, 3)	0.94 (0.33, 4)

^a^ data converted from [16].

## Data Availability

The original contributions presented in this study are included in the article. Further inquiries can be directed to the corresponding author.

## Abbreviations

The following abbreviations are used in this manuscript:

AA	Antimycin A
ADP	Adenosine diphosphate
A_F_	2-Oxoadipate dehydrogenase (flavoprotein)
ANOVA	Analysis of variance
ATP	Adenosine triphosphate
AUR-HRP	Amplex UltraRed–horseradish peroxidase
B_F_	Branched-chain 2-oxoacid dehydrogenase (flavoprotein)
BSA	Bovine serum albumin
CI	Complex I
CII	Complex II
CIII	Complex III
CIV	Complex IV
EGTA	Ethylene glycol-bis(β-aminoethyl ether)-N,N′-tetraacetic acid
EC_50_	Half maximal effective concentration
ETC	Electron transport chain
GRX2	Glutaredoxin-2
H_2_O_2_	Hydrogen peroxide
IC_50_	Half maximal inhibitory concentration
I_F_	Complex I flavin site
II_F_	Complex II flavin site
III_Qi_	Inner Q-binding site of Complex III
III_Qo_	Outer quinol-binding site of Complex III
I_Q_	Complex I ubiquinone site
MIB	Mitochondrial isolation buffer
MRB	Mitochondrial respiration buffer
NA	Naphthenic acid
NAFCs	Naphthenic acid fraction compounds
NAD^+^	Nicotinamide adenine dinucleotide (oxidized form)
NADH	Nicotinamide adenine dinucleotide (reduced form)
NSERC	Natural Sciences and Engineering Research Council of Canada
O_F_	2-Oxoglutarate dehydrogenase (flavoprotein)
OGDH	2-Oxoglutarate dehydrogenase complex
ON	Ontario
OSPW	Oil sands process-affected water
OXPHOS	Oxidative phosphorylation
P_F_	Pyruvate dehydrogenase (flavoprotein)
QC	Quebec
Q/QH2	Ubiquinone/ubiquinol pool
ROS	Reactive oxygen species
SEM	Standard error of the mean
Tris HCl	Tris hydrochloride
Δψ_m_	Mitochondrial membrane potential
